# Defining “Giant” Mediastinal Tumors: Proposal of a New Clinical–Radiological Classification and Case Report

**DOI:** 10.3390/diagnostics15020159

**Published:** 2025-01-12

**Authors:** Emanuel Palade, George Bucur Delaca, Ioana-Medeea Titu, Sergiu Adrian Ciulic, Gabriel Cismaru, Adrian Stef, Simona Manole

**Affiliations:** 1 Department of Surgery, Iuliu Hatieganu University of Medicine and Pharmacy, 400000 Cluj-Napoca, Romania; paladeemanuel1@gmail.com (E.P.);; 2 Thoracic Surgery Clinic, Leon Daniello Clinical Hospital of Pneumology, 400371 Cluj-Napoca, Romania; 3 4th Department of Internal Medicine, Iuliu Hatieganu University of Medicine and Pharmacy, 400347 Cluj-Napoca, Romania; 4 Cardiac Rehabilitation Clinic, Rehabilitation Hospital, 400347 Cluj-Napoca, Romania; 5 Niculae Stancioiu Heart Institute, Calea Motilor 19-21, 400001 Cluj-Napoca, Romania; 6 Department of Radiology and Medical Imaging, Iuliu Hatieganu University of Medicine and Pharmacy, 400012 Cluj-Napoca, Romania

**Keywords:** giant mediastinal tumors, thymolipoma, mediastinal mass effect

## Abstract

**Background/Objectives:** Mediastinal tumors, regardless of their location, can grow to significant sizes, causing compression-related symptoms. The term “giant” mediastinal tumor is inconsistently defined in the literature. This study presents a new clinical–radiological classification (CRC) for mediastinal tumors and evaluates its applicability through a systematic review and a detailed case analysis of a giant thymolipoma. **Methods**: A systematic review of the literature from the past decade was conducted using PubMed to identify relevant studies on “giant” mediastinal tumors. The inclusion criteria focused on studies involving adult patients with documented tumor size and symptomatology. The review identified 22 studies, with most anterior mediastinal tumors classified as CRC 3 (81%), indicating “giant” tumors. Thymolipomas accounted for 58% of these cases. Tumor volume and weight correlated with symptom severity, guiding surgical approaches. The proposed CRC effectively standardized the definition of “giant” tumors. The case analysis of a 6.84 kg thymolipoma highlighted the challenges of surgical resection, confirming the importance of tailored surgical strategies for large tumors. **Results:** The review of the literature revealed a significant variation in tumor size and weight across the different mediastinal compartments. Symptomatic tumors (CRC stage 3) located in the anterior mediastinum exhibited the largest volumes and weights, with an average volume of 4949 mL (range: 2013–8840 mL) and an average weight of 4137 g (range: 1575–7500 g). In comparison, tumors in the posterior mediastinum ranked second in terms of size, with an average volume of 2128 mL (range: 1040–5460 mL) and an average weight of 2489 g (range: 1009–6000 g). Tumors located in the middle mediastinum were considerably smaller, with an average volume of 536 mL (range: 21–1092 mL). Among the largest symptomatic tumors in the anterior mediastinum, thymolipomas were the most frequently observed histologic type. These findings underscore a clear size gradient across the mediastinal compartments, with the anterior mediastinum harboring the largest symptomatic tumors, followed by the posterior mediastinum, and the smallest tumors in the middle mediastinum. **Conclusions**: The novelty of the study lies mainly in the new clinical–radiological classification (CRC) of mediastinal tumors. This classification integrates clinical presentation and cross-sectional imaging findings, offering a standardized framework for tumor reporting. In addition, it provides a precise definition of “giant” mediastinal tumors. The findings emphasize the need for early surgical intervention to prevent severe symptoms and complications. This study also showcases the largest *enbloc*-resected thymolipoma reported in the recent literature, supporting the utility of the proposed classification in clinical practice.

## 1. Introduction

Mediastinal tumors, regardless of their origin in one of the three mediastinal compartments (anterior, middle, or posterior, according to the International Thymic Malignancy Interest Group—ITMIG), can reach large volumes and can eventually produce symptoms, due to the compression of surrounding anatomical structures [1]. The term “giant” mediastinal tumor is commonly used to describe such cases, despite the fact that this term was never clearly defined. According to a comprehensive review of the histologic type of “giant” mediastinal tumors (tumors larger than 10 cm in diameter, defined by the authors) published in 2023, these tumors can be classified into mesenchymal tumors, thymic tumors of epithelial origin, and germ cell tumors. Among the mesenchymal tumors, adipocytic tumors (thymolipoma, liposarcoma, and thymoliposarcoma), although rare, are known for their potential to reach large sizes and produce local symptoms [2].

Thymolipomas are rare (approximately 2–9% of all thymic tumors), benign tumors originating in the anterior mediastinum, composed predominantly of mature adipose tissue interspersed with islands of thymic tissue [3]. Thymolipomas can occur at any age, affect mainly young adults in their third decade, and are equally prevalent among men and women [3,4]. While often asymptomatic, they can grow to significant sizes, leading to symptoms related to the compression of adjacent anatomical structures [5]. Common symptoms include dyspnea, chest pain, and a cough, but cardio-circulatory impairment, dysphagia, or even abdominal pain may also occur. The intensity of the symptoms depends on the size, location, and extent of the tumor. Up to 40% of thymolipomas are associated with autoimmune disorders such as myasthenia gravis (most frequent), hypogammaglobulinemia, aplastic anemia, or vitamin B12 deficiency [3].

For diagnosis, computed tomography (CT) scanning is particularly useful for identifying the adipose nature of the tumor and thymolipomas presenting as well-defined masses with low attenuation values consistent with fat. Contrast-enhanced CT studies facilitate the detailed characterization of the mass’s vascularization and identify areas of compression on adjacent mediastinal structures, making them indispensable for surgical planning. Magnetic resonance imaging (MRI) further aids in differentiating thymic tissue from adipose components [5,6].

Thymolipomas, like other primary mediastinal tumors (excluding lymphomas), are addressed by surgery. Complete excision is usually curative, with a low risk of recurrence. The surgical approach depends on the tumor’s size and location. Midline sternotomy is commonly employed for large tumors, providing adequate exposure for complete excision. Thoracotomy, or even minimally invasive techniques such as video-assisted thoracoscopic surgery (VATS), have also been used successfully in selected cases [7]. Ferrari et al. described the use of bilateral sequential video-assisted thoracoscopy to excise a large thymolipoma by dividing it into two parts, highlighting the benefits of minimally invasive surgery for suitable patients [4]. After removal, significant symptom relief occurs and operative complications, including bleeding, incomplete lung re-expansion, prolonged air leak or arrhythmias, are rare [5,8,9].

In general, so called “giant” mediastinal tumors are reported to produce symptoms related to the compression of neighbored organs. Although the term “giant” is not clearly defined, it is used to describe very large masses, and only two publications have attempted to address this issue and offer a definition. First, Collaud, S. et al. (2023) presented their experience of middle mediastinal masses (*n* = 36) and defined a giant middle mediastinal tumor as having a size equal to or exceeding the 90th percentile (73 mm in diameter) of the tumors in the analyzed cohort. Interestingly, all four tumors identified as giant were mesenchymal (esophageal leiomyoma, synovial sarcoma, leiomyosarcoma, and round cell sarcoma) in nature, despite the extreme rarity of these tumors [10]. Second, Brcic, L. et al. (2023) reviewed the literature on the histopathologic features of giant mediastinal tumors, using, for research purposes, a size of at least 10 cm in largest tumor diameter to define “giant” lesions. The authors admit that this cutoff was chosen arbitrarily [2].

The main objective of this study is to propose a new clinical–radiological classification (CRC) of mediastinal tumors. These masses, depending on their volume and location, can cause compression-related symptoms. Additionally, this study aims to highlight the particularities of large (“giant”) mediastinal masses regarding clinical presentation and surgical management through a literature review. The CRC also offers objective criteria for defining the term “giant”, as this has been widely used without a clear definition. From a practical perspective, defining this term has implications in clinical management, as “giant” mediastinal tumors can cause severe or even life-threatening compression-related symptoms. Furthermore, the surgical removal of “giant” tumors requires complex procedures that are well planned and performed in order to minimize operative morbidity and mortality. The novelty of our study mainly consists of offering this new clinical–radiological classification of mediastinal tumors, which can help to standardize the terminology used to report on mediastinal tumors and further aid clinicians in surgical decision-making for these complex cases.

## 2. Materials and Methods

**Figure 1:**
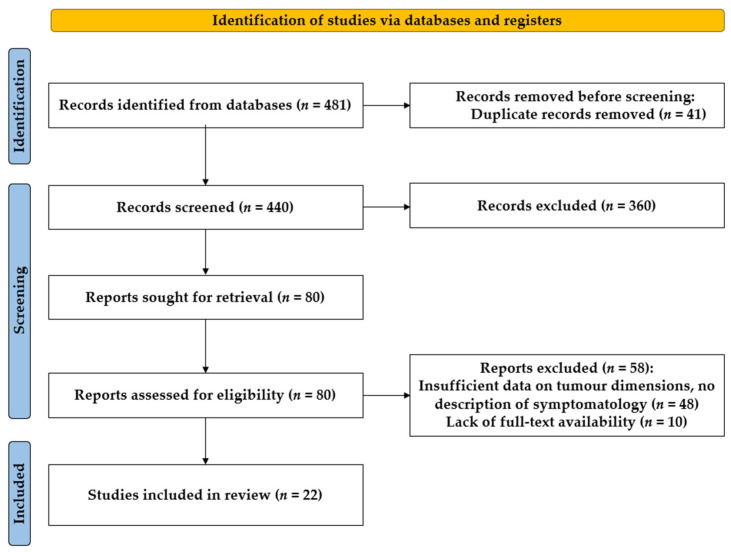
PRISMA diagram showing the results of the literature search.

A systematic review of the literature from the past 10 years was conducted, including studies published in English with full-text versions available and indexed in PubMed. The following keywords were used: “thymic lipoma”, “thymic tumors”, “giant mediastinal tumors”, “giant thymic tumors”, and “thymolipoma”. Case reports, case series, and clinical studies were included in the review. To ensure relevance, studies providing data on tumor mass and dimensions were selected. Additionally, only cases involving adult patients were included. Mediastinal lymphomas, which are not treated with surgical resection, and tumors associated with paraneoplastic syndromes, such as thymomas producing myasthenia gravis, were excluded from the analysis. Our study may be subject to potential biases due to limitations in the search criteria. This approach was chosen to ensure high-quality, complete, and transparent data. The data collection process was performed manually, relying on a structured and consistent approach to identify and evaluate relevant studies. However, this may have excluded relevant studies published in other languages or outside the selected timeframe, potentially limiting the generalizability of our findings. Figure 1 illustrates the search results using the PRISMA diagram.

Due to the absence of a standardized classification system based on the size of mediastinal tumors and the widespread use of subjective descriptors (“large”, “giant”, “voluminous”, and “massive”), we propose a clinical–radiological classification (CRC) of mediastinal tumors. The classification takes into account the clinical observation that both benign and malignant mediastinal tumors are often slow-growing masses that can reach significant volumes without producing local symptoms. Since there is only a loose correlation between tumor size and symptom severity, the classification is based on both cross-sectional imaging findings and clinical manifestations related to the compression of adjacent anatomical structures. Symptoms related to paraneoplastic manifestations were excluded, as tumors producing such symptoms are typically diagnosed before reaching large volumes. Using this classification, the term “giant mediastinal tumor” is now objectively defined. Table 1 shows the proposed clinical–radiological classification (CRC) of mediastinal tumors.

According to our proposed clinical–radiological classification, “giant” mediastinal tumors are defined as those causing both the compression of adjacent anatomical structures (visible on imaging) and compression-related symptoms. To validate this definition and explore the distinct features of giant mediastinal tumors, we analyzed parameters such as symptoms, tumor size, tumor weight, tumor volume, histology, surgical approach, and operative complications. Based on the information provided in the publications reviewed, a CRC stadium was attributed to each case. Since the mass effect of a tumor is related to its dimensions and a comparison between sizes is challenging, tumor volume was used for analysis. This parameter was approximated using the following ellipsoid volume formula: volume (mL) = height (cm) × length (cm) × width (cm) × 0.52.

Additionally, we present a recent case of a giant thymolipoma treated in our department, to illustrate these findings and demonstrate the application of our proposed classification.

## 3. Results

The literature review of the last 10 years on “giant” mediastinal tumors regarding all three anatomic compartments of the mediastinum revealed a total of 22 studies (15 on the anterior, 2 on the middle, and 5 on the posterior mediastinum). The results of this search are presented in Table 2 [7,8,9,10,11,12,13,14,15,16,17,18,19,20,21,22,23,24,25,26,27,28].

For the anterior mediastinum, 3 studies (Kaplan T, 2014, Daniel VC, 2023 and Soleimani H, 2024 [11,21,22]) reported that the patients were asymptomatic, while the remaining 12 studies offered comprehensive data on the symptoms related to the mediastinal mass. The predominant symptom reported was dyspnea, varying in severity (in one case with emergency presentation due to dyspnea) and generally progressive over time. Other symptoms included a cough, chest pain, and weight loss. Regarding the CRC stadium, the majority (81%, *n* = 13) were classified as CRC 3 (9 cases CRC 3A and 4 cases CRC 3B), justifying the use of the term “giant” to characterize these tumors.

Among the 16 tumors reported in the anterior mediastinum, the most frequent histologic type was thymolipoma (*n* = 10), with 2 cases associated with thymoma. Other histologic types included lipoma (*n* = 2), liposarcoma (*n* = 3), and thymoma (*n* = 3, with 2 associated with thymolipoma).

Anterior mediastinal tumors producing symptoms (stadium CRC 3) had an average volume of 4949 mL (range: 2013–8840 mL) and an average weight of 4137 g (range: 1575–7500 g), significantly larger than asymptomatic anterior mediastinal masses (volume 1450 mL, range 468–2332 mL, weight 1273 g, range 1060–1485 g). Moreover, thymolipomas (associated with thymoma or not) exhibited similar average volumes and weights (4300 mL, range: 468–8840 mL and 3857 g, range: 1060–7500 g, respectively) to other anterior mediastinal tumors (4281 mL, range: 2013–7800 mL and 3601 g, range: 1575–5400 g, respectively).

The surgical approach for tumor excision was median sternotomy in 7 cases (extended by anterolateral thoracotomy in 2 cases), clamshell incision in 1 case, and thoracotomy in 7 cases (in 1 case bilateral posterolateral thoracotomy). One report did not mention the surgical approach. The tumors were *enbloc* resected in 12 cases, divided into 2 components in 2 cases, and via piecemeal resection in another 2 cases.

Among the 13 resections with reported postoperative morbidity, 77% (*n* = 10) had an uneventful recovery and 3 patients (23%) experienced complications such as intraoperative bleeding, respiratory insufficiency necessitating mechanical ventilation, and incomplete re-expansion of the tumor-compressed lung.

For the middle mediastinum, only two reports on “giant” tumors, with a total of five cases, were found. Except for one case, all patients had symptoms such as a cough, dyspnea, and chest pain. One patient with the tumor mass producing anterior displacement of the heart and right pulmonary artery, and compressing the esophagus and both main bronchi, causing worsening chest pain, was delivered to the emergency department. The majority (80%) were classified as stadium CRC 3 (giant tumors), despite their much smaller average volume (average 536 mL, range: 21–1092 mL) compared to anterior mediastinal giant tumors. All tumors were mesenchymal, with two benign tumors (solitary fibrous tumor and esophageal leiomyoma) and three (60%) sarcomas (synovial sarcoma, leiomyosarcoma, and undifferentiated round cell sarcoma). Surgical data were available in four cases. The preferred surgical approach was posterolateral thoracotomy (three cases out of four reported), with one case involving a median sternotomy for an extended transpericardial middle mediastinal mass resection under cardiopulmonary bypass. Despite the complexity of the surgical procedures, none of the patients experienced operative complications.

Regarding the posterior “giant” mediastinal tumors, five reports were found, all providing complete data on symptoms, histology, tumor size, and weight. Dyspnea was a constant symptom, often associated with a dry cough, dysphagia, chest pain, weight loss, and atrial flutter. All cases were CRC 3, with two cases presenting in the emergency department—one with acute respiratory failure requiring intubation and mechanical ventilation, and the other with atrial flutter. Histologically, liposarcoma was encountered in three cases (60%) and schwannoma in two. The average volume of the masses was 2128 mL (range: 1040–5460 mL) and the average weight was 2489 g (range: 1009–6000 g). All tumors were completely *enbloc* resected via thoracotomy, both anterolateral and posterolateral. Except for one case requiring reintubation and mechanical ventilation over 11 days due to pneumonia, all cases had an uneventful postoperative recovery.

For a better understanding of the proposed classification and its application, we present a recent case of a giant thymolipoma, originating in the anterior mediastinal compartment and extended into both pleural cavities.

**Figure 2:**
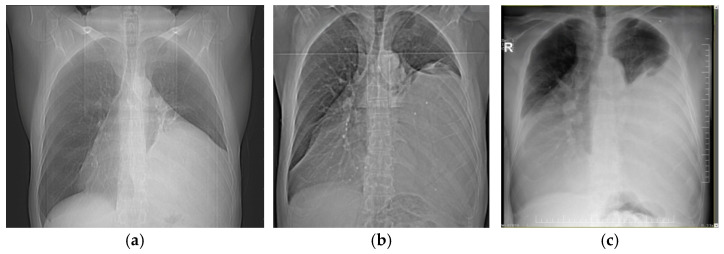
Conventional chest radiography of the patient from the presentations in 2008 (**a**), 2018 (**b**), and 2023 (**c**) showing significant growth over time of the dense opacity caused by the tumor, with massive compression of both lungs and mediastinal shift towards right hemithorax.

**Figure 3:**
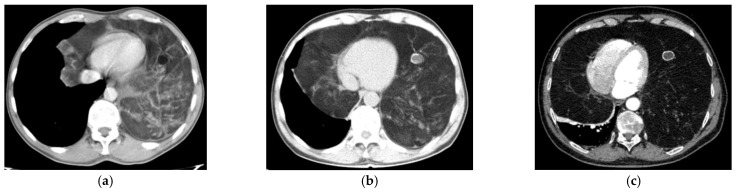
CT scan from the same presentations in 2008 (**a**), 2018 (**b**), and 2023 (**c**), revealing the large mediastinal mass consisting of inhomogeneous tissue with fatty densities, fibrous trabeculae, and intratumorally calcification.

A 53-year-old male patient, initially presenting in 2008 with conventional chest radiography to investigate a respiratory infection, was found to have a large opacity in the left lower pulmonary field. A CT scan revealed a large mass with fatty tissue content occupying the lower third of the left hemithorax and extending retrosternally into the right paracardial region. Based on the imaging, a CRC stadium 2 can be presumed at the time of the initial encounter. The patient declined the proposed surgical intervention. Ten years later (2018), he presented again with dyspnea upon exertion, and intermittent chest discomfort, as well as a significantly larger tumor mass extending into both the left and right hemithorax and causing significant lung compression (stadium CRC 3A). In 2023, the patient returned with worsening dyspnea at rest, a major decrease in exercise capacity (50-m walking distance), atrial fibrillation, and a significant increase in the mediastinal mass visible on CT scan (stadium CRC 3B). Recent imaging revealed a very large mass measuring 50 × 30 × 15 cm, predominantly located in the left hemithorax and extending precordially into the right hemithorax, causing a mediastinal shift towards the right and the compression of both lungs. The patient’s medical history also included hypertension, left ventricular insufficiency, hepatitis B, and thrombocytopenia. Figure 2a–c and Figure 3a–c illustrate the conventional radiological and CT appearances of tumor growth over time, from 2008 to 2018 and 2023.

Given the tumor’s significant size and the patient’s worsening symptoms, surgical intervention was planned. Dynamic analysis allowed for careful preoperative planning (approach and intraoperative strategy), identifying the tumor’s primary origin in the left hemithorax and therefore anticipating that the vascular supply would arise predominantly from the left side.

**Figure 4:**
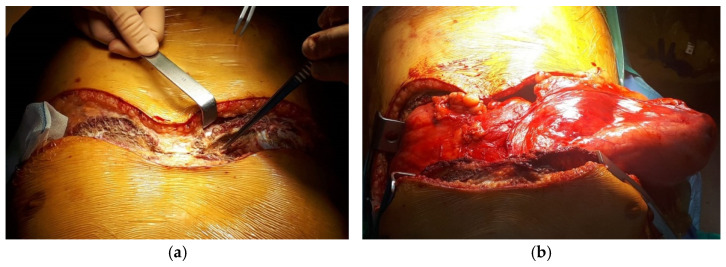
Clamshell incision (**a**) and first step of the tumor resection by mobilizing the right-sided component (**b**).

**Figure 5:**
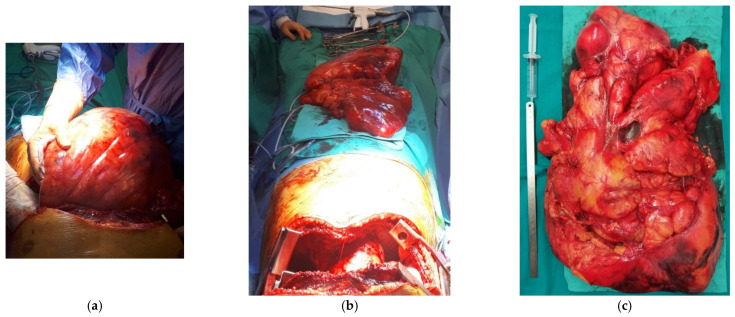
End stage of the tumor resection in the left hemithorax (**a**), chest cavity after tumor removal (**b**), and operative specimen (**c**).

A clamshell incision was made in the fourth intercostal space to provide optimal exposure and facilitate the complete removal of the tumor (Figure 4a). The resection began with the right-sided component of the tumor (Figure 4b). The tumor, measuring 50 × 30 × 15 cm and 6.84 kg in weight (Figure 5c), was found to be supplied predominantly by the left internal mammary artery. It consisted of encapsulated, polylobate adipose tissue, without infiltration into the surrounding structures, although there were dense adhesions to the surface of the left lung. The tumor was *enbloc* resected, starting from the right hemithorax to optimize exposure of the larger left-sided component. Both phrenic nerves remained intact, and no resection of adjacent anatomical structures was necessary. Despite the normalization of the heart position after resection, the compressed left lung failed to fully expand and occupy the entire left pleural cavity (Figure 5a–c).

**Figure 6:**
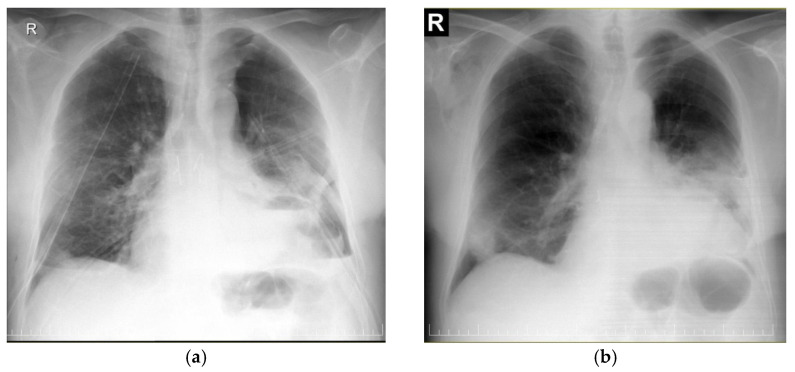
Conventional chest radiography on postoperative day 4 with bilateral pleural drainage, and small left basal pneumothorax still present, as the left lung initially could not re-expand fully (**a**), and at discharge with closed left pleural space by smaller left lung and elevated left hemidiaphragm (**b**).

The postoperative period was complicated by recurrent high-rate atrial fibrillation with significant hemodynamic impact, requiring antiarrhythmic therapy (Amiodarone) for heart rate control. Typical surgical complications were absent, except for a prolonged air leak requiring left-sided chest drainage for 12 days. The patient was discharged on postoperative day 18 in a good condition, and had fully recovered one month later without further complications (Figure 6a,b).

Pathological examination of the resected mass confirmed the diagnosis of a benign thymolipoma, composed of mature adipose tissue, islands of thymic tissue, and scattered myoid cells.

## 4. Discussion

In a relatively confined anatomic region filled with vital anatomical structures like the mediastinum, the volume of a tumor that develops in this area has an important impact, not only on the type and severity of the symptoms, but also on the surgical approach required for its resection. On rare occasions, these tumors can reach impressive dimensions, making surgical resection particularly challenging in terms of approach, exposure, and potential operative complications. When such cases are reported in the literature, in order to underline the particularities and difficulties encountered, the authors refer to these masses as being “giant”, “voluminous”, “large”, “massive”, or even “huge”. However, these terms are subjective and lack clear definitions, making it difficult to objectively categorize such tumors and meaningfully compare findings across reports. We encountered this issue while preparing to report on a recent case of a very large thymolipoma that was surgically removed in our department.

To address this lack of definition, we propose a classification system for mediastinal tumors based on their size and the clinical–radiological findings. Our classification, termed the “clinical–radiological classification” (CRC), is grounded in the observation that tumors generally produce local symptoms only when they reach a certain degree of compression. Additionally, there is a loose correlation between tumor size and the intensity of symptoms. In this classification, “giant” mediastinal tumors are now clearly defined as those producing both the deformation of surrounding anatomical structures, visible on imaging, and symptoms (stadium CRC 3A with mild to moderate symptoms and stadium CRC 3B with severe symptoms, up to conditions addressed as emergencies). To analyze the value of this new classification, we performed a search of the literature of the last 10 years on “giant” mediastinal tumors, and compared the different reports, as well as the particularities of the tumors located in different mediastinal compartments (as defined by ITMIG) [1]. For the anterior mediastinum, we additionally presented our case and its challenges.

When the CRC is applied to the results of our search for “giant” mediastinal tumors, several aspects emerge. For the anterior mediastinal compartment, most of the tumors reported (81%, *n* = 13) were classified as “giant”, according to our criteria. There was also a significant difference of about 3.5 L and 2.9 kg, in the average volume and weight, between CRC 3 (“giant” mediastinal tumors) and all other tumors (stadium CRC 1 and CRC2), giving the term “giant” a more accurate significance. Among the four tumors with severe symptoms (CRC 3B), representing 31% of the giant anterior mediastinal tumors, one case required emergency intervention due to severe dyspnea. Histologically, the majority of CRC 3 tumors were adipocytic (92%), with thymolipoma representing about 58% of those tumors. The only other histologic type of CRC 3 tumor reported was thymoma, in one case. Although thymolipomas and the other adipocytic tumors are frequently reported as giant tumors, thymolipomas are not necessarily the largest, as a comparison of thymolipomas with all other tumors combined (regardless of CRC stadium) showed similar average values for the volume and weight (4300 mL vs. 4281 mL and 3857 g vs. 3601 g, respectively).

As all analyzed cases referred to surgically removed tumors, a critical issue is related to surgical approach. As tumor size increases, particularly when the tumor extends into the pleural cavities, exposure—especially to the posterior aspects of the tumor—becomes progressively more difficult.

In all CRC 3 tumors (*n* = 13), the median sternotomy (*n* = 7) and clamshell incision (*n* = 1) allowed for *enbloc* resection, except for in one case where a two-component resection was necessary. In this latter case, the exposure had to be improved by adding a right anterior thoracotomy, as significant intraoperative bleeding (transfusion of 3 units of blood) occurred. The authors noted that “using this approach, we managed to perform the operation in less time, and it was easier to detect the right phrenic nerve and the right lung hilum while causing less bleeding than with the median sternotomy approach alone” [8]. This observation supports our recommendation that the surgical approach should be large enough and tailored to the tumor’s size to ensure optimal exposure. In our case of giant thymolipoma, the clamshell incision was planned and performed from the beginning, as the tumor had largely extended into both pleural cavities. One other tactical aspect we recommend is to initiate the tumor resection with the smaller tumor component (the right hemithorax, in our case), to improve the exposure of the larger component. This strategy aids in controlling the tumor’s contact areas with adjacent organs, particularly vascular pedicles. This observation regarding the impact of an appropriate approach seems to be supported by the fact that out of the five CRC 3 tumors resected via thoracotomy, *enbloc* resection was possible in only two cases. In another two cases, a piecemeal resection was performed, and in the last case, a two-component resection was performed using a bilateral posterolateral thoracotomy. In this last case, the authors concluded that “a clamshell approach generally allows the best surgical field of the anterior mediastinum and the thoracic cavity” [12]. Furthermore, a complete *enbloc* resection should be the surgical goal, even in benign-appearing tumors. For instance, thymolipomas can contain thymomas, as reported by Kaplan, T. et al. and Daniel, V.C. et al., and lipomas are difficult to distinguish from well-differentiated liposarcomas, making the use of the frozen section to confirm benignity prior to a piecemeal resection ineffective [2,11,21]. Our review indicates that the thymolipoma we removed was the largest *enbloc*-resected mediastinal tumor reported in the past 10 years, surpassing the 7.5 kg piecemeal resection of a thymolipoma by Gong, L.H. et al. [9].

Regarding the complications, intraoperative bleeding with a need for blood transfusion was reported in two (15.4%) out of thirteen cases where complications were reported. In one other case, respiratory insufficiency necessitating mechanical ventilation and incomplete re-expansion of the tumor-compressed lung complicated the operation. All reported complications (bleeding, respiratory insufficiency, and incomplete lung re-expansion) are expected events in the context of large mediastinal tumors. Therefore, a complication rate of 23% is considered acceptable. In our case, the compressed left lung initially could not fully re-expand, and prolonged chest drainage over 12 days was mandatory due to a persistent air leak. In addition, the preexisting atrial fibrillation persisted, but was controlled with antiarrhythmic therapy.

For the middle mediastinum and posterior mediastinum, the number of cases that met our inclusion criteria was much smaller, limiting our ability to perform a comprehensive analysis. Thus, all five cases presented in the two reports had mesenchymal tumors, with only two of them (40%) being benign and the rest sarcomas. Using the CRC, 80% were classified as CRC 3 (giant mediastinal tumors), presenting respiratory symptoms such as a cough, dyspnea, and chest pain, despite having a much smaller average volume (536 mL) than the tumors in the anterior mediastinum. This finding may be explained by the more intimate relationship between the tumor and the anatomical structures of the visceral compartment. The preferred surgical approach for the middle mediastinum was the posterolateral thoracotomy, with only one case requiring a median sternotomy, due to suspected cardiac wall involvement. No operative complications were noted, not even in the challenging resections of sarcomas.

For the posterior mediastinum, five cases were reviewed, all of which were classified as CRC 3. All patients presented dyspnea in association with other symptoms such as a cough, chest pain, dysphagia, weight loss, and atrial flutter. Two patients presented as emergencies, one with acute respiratory failure requiring emergency intubation and the other with atrial flutter. With an average tumor volume of 2128 mL and weight of 2489 g, the tumors in the posterior mediastinum occupy the place between the anterior mediastinal and the middle mediastinal masses, suggesting that the compression effect appears more slowly as for middle mediastinal tumors, but more rapidly as for anterior mediastinal masses. Although neurogenic tumors are common in this location, only two schwannomas were reported as giant tumors, the rest (60%) being liposarcomas. As with the middle mediastinum tumors, the preferred surgical approach is lateral thoracotomy, allowing for all presented giant posterior mediastinal tumors to undergo a complete *enbloc* resection. Except for the patient intubated in the emergency room for acute respiratory failure, which postoperatively developed into pneumonia requiring 11 days of mechanical ventilation, all other patients had an uneventful recovery.

Regarding the mediastinal tumors producing local symptoms (CRC 3), our literature review showed that tumors in the anterior mediastinum can reach much larger volumes until producing compression-related symptoms, compared to those in the posterior and especially in the middle mediastinum. Tumors in these latter compartments, due to their proximity to vital structures in the visceral compartment, tend to produce symptoms earlier on.

Attempts to offer a definition of “giant” mediastinal tumors based exclusively on size were proposed by only two authors. The 10 cm cut off for maximal tumor diameter was used by Brcic, L. et al., only to have a reference for the literature search for their review on the histopathological features of giant mediastinal tumors. As in our review, hematolymphoid neoplasms were excluded, as these tumors are not subject to surgical resection, although these masses can reach large volumes [2]. Compared to the proposed CRC, the 10 cm cut off does not correlate properly with the CRC 3 stadium (giant tumor). As for the anterior and posterior mediastinal tumors, the sizes are, in general, much larger, and for the middle mediastinal tumors, they are smaller than 10 cm. The second definition of “giant” mediastinal tumors was proposed by Collaud, S. et al., based on their experience of thirty-six patients operated upon for middle mediastinal masses. The authors defined a “giant” middle mediastinal tumor as having a diameter equal to or larger than the 90th percentile of the distribution of tumor diameters (73 mm). Four tumors out of thirty-six (11%) were larger than 73 mm [10]. Applying the CRC to the tumors reported by Collaud, S. et al. (see Table 2), one tumor was stadium CRC 1, despite a diameter of 82 mm, and the other three were classified as CRC 3A [10].

The proposed (CRC) of mediastinal tumors offers an objective method for categorizing mediastinal masses, particularly large ones (“giant” tumors), which pose significant surgical challenges in terms of exposure and protecting the surrounding vital organs, but also regarding the selection of an appropriate surgical approach. For the anterior mediastinal masses extended into one or both pleural cavities, the lateral approach using thoracotomy, or even a median sternotomy, can be insufficient, and can lead to compromises such as performing a piecemeal resection. Therefore, in such cases, we advocate for the use of a hemiclamshell or a clamshell incision, depending on the tumor extension, from the anterior mediastinum in one or both pleural spaces. But also, for the middle and posterior mediastinal tumors, which are addressed by thoracotomy, depending on the CRC stage, one can choose an anterolateral thoracotomy for CRC 1 and 2 tumors, and a posterolateral thoracotomy for CRC 3 masses.

Since the CRC is based on clinical and radiological features, it can be applied not only to all three mediastinal compartments, but also to other anatomical regions where large tumors may occur and produce compression-related symptoms, such as the abdominal cavity (especially the pelvic region and retroperitoneum). Additionally, the proposed CRC may prove particularly valuable in the pediatric population, as children are more susceptible to developing symptoms from tumor compression due to their smaller anatomical spaces and developing physiology. Future research should investigate its utility in this population.

In terms of limitations, our study is constrained by the retrospective nature of the analysis and the rarity of the pathologies presented. These factors influenced our study design, necessitating reliance on a comprehensive literature review to validate the proposed CRC. Additionally, the biases already mentioned, such as language and time-frame restrictions, further limit the generalizability of our findings. Despite these limitations, the CRC serves as the central innovation of this study, offering a novel framework for understanding and managing mediastinal tumors.

## 5. Conclusions

Mediastinal tumors, both benign and malignant, can reach considerable volumes and produce compression-related symptoms. When reported in the medical literature, the authors frequently refer to these masses using subjective terms such as “giant”, “voluminous”, “large”, or “huge”. The proposed clinical–radiological classification (CRC), based on cross-sectional imaging findings and compression-related symptoms, provides a standardized and objective framework for describing and categorizing mediastinal tumors. Specifically, the clear definition of “giant” mediastinal tumors offers a practical tool for clinical practice and research. Surgical resection is the treatment of choice, and the use of an adequate approach to obtain optimal exposure is mandatory to obtain a complete *enbloc* resection, and to reduce the risk of operative complications. The operative removal of mediastinal masses is mandatory prior to the development of local symptoms, to avoid disabling symptoms and challenging surgical procedures. Additionally, the most frequent reported giant tumor (stadium CRC 3A or 3B) in the anterior compartment seems to be thymolipoma. The case presented underlines the practical value of the CRC, offers arguments for the surgical strategy used, and supports the recommendation of an early surgical removal of these masses, as they can evolve over time, producing severe symptoms. The novelty of our study lies especially in the proposed CRC of mediastinal masses and the presentation of the best documented case of a giant thymolipoma over a long period of time, which in addition is the largest *enbloc*-resected thymolipoma published in the last 10 years.

## Figures and Tables

**Table 1:** Proposed clinical–radiological classification (CRC) of mediastinal tumors.

CRC Stadium	Clinical-Radiological Findings
CRC 1 (minimal compression)	- minimal lung compression, no mediastinal shift or deformation of mediastinal structures observed on cross-sectional imaging (CT/MRI) - asymptomatic patients
CRC 2 (moderate compression)	- mediastinal shift, compression of the lung and/or mediastinal structures evidenced by cross-sectional imaging (CT/MRI) - asymptomatic patients
CRC 3 (“giant mediastinal tumor”)	
CRC 3A	- mediastinal compression with significant shift and deformation of adjacent structures - mild to moderate dyspnea chest discomfort
CRC 3B	- mediastinal compression with marked shift and deformation of adjacent structures - severe, debilitating symptoms (severe dyspnea, superior vena cava syndrome, dysphagia, cardiac insufficiency or arrhythmias)

**Table 2:** Results of literature review on “giant” mediastinal tumors for all three mediastinal compartments (* CRC stadium was appreciated based on the information found in the reviewed text; ^#^ tumor volume was calculated using the ellipsoid formula based on the size values offered in the reviewed text).

Study	Study Type	Tumor Localization (Mediastinal Compartment)	Sex	Age (y)	Symptoms	CRC Stadium *	Histology	Size (cm)	Weight (gr)	Volume (mL) ^#^	Surgical Approach	Operative Complications
Kaplan, T. et al., 2014 [11]	case report	anterior	female	23	no symptoms	CRC 1	thymolipoma and B1 thymoma	20 × 15 × 3 cm	1485	468	right lateral thoracotomy *enbloc* resection	not mentioned
Pei, G. et al., 2015 [12]	case report	anterior	female	30	shortness of breath for approximately 3 years, with exacerbation in the last 3 months	CRC 3B	thymolipoma	1. 20 × 17 × 15 cm 2. 28 × 25 × 17 cm	4150	8840	double posterolateral thoracotomy tumor resected in two components	none
Vaziri, M. et al., 2016 [13]	case report	anterior	male	40	progressive dyspnea and cough over the past 10 months	CRC 3A	thymolipoma	40 × 33 × 8 cm	4000	5491	left posterolateral thoracotomy piecemeal resection	none
Goswami, A. et al., 2017 [14]	case report	anterior	male	68	progressively increasing effort intolerance and fatigue for 4 months	CRC 3A	thymolipoma	25 × 20 × 12 cm	3200	3120	median sternotomy *enbloc* resection	respiratory insufficiency (mechanical ventilation for 3 days)
Aghajanzadeh, M. et al., 2018 [15]	case report	anterior	male	35	progressive chest pain, cough, dyspnea, and a right-sided neck swelling for 2 years, aggravated in the last 2 months	CRC 3B	thymolipoma	31 × 21 × 8 cm	5000	2708	right extensive posterolateral thoracotomy *enbloc* resection	none
Yang, Y.S. et al., 2018 [16]	case report	anterior	male	63	chronic cough and progressive dyspnea upon exertion	CRC 3A	liposarcoma	22 × 16 × 11 cm	1575	2013	left thoracotomy *enbloc* resection	none
Chen, C. et al., 2018 [17]	case series (2 cases)	anterior	female	22	mild dyspnea upon exertion	CRC 3A	lipoma	40 × 25 × 15 cm	3780	7800	median sternotomy *enbloc* resection	none
			male	43	gradually progressive dyspnea over 4 months	CRC 3A	liposarcoma	28 × 25 × 10 cm	2850	3640	median sternotomy *enbloc* resection	none
Nguyen et al., 2018 [18]	case report	anterior	female	48	shortness of breath, dyspnea upon exertion, progressive nonproductive cough, and weight loss	CRC 3B emergency presentation due to dyspnea	liposarcoma	27 × 27 × 9 cm	3500	3411	clamshell thoracotomy *enbloc* resection	none
Sharma, KC. et al., 2019 [19]	case report	anterior	male	23	cough and dull ache pain for 4 weeks	CRC 3A	thymolipoma	26 × 18 × 11 cm	6000	2676	anterolateral thoracotomy and sternotomy *enbloc* resection	none
Daoud, D. et al., 2021 [8]	case report	anterior	female	18	progressive exertional dyspnea, weight loss, and loss of appetite	CRC 3A	thymoma B1	36 × 29 × 10 cm	4500	5428	median sternotomy extended with right-sided hemiclamshell tumor resected in two components	intraoperative bleeding and transfusion of 3 units blood
Siddiqi, M.S. et al., 2021 [7]	case report	anterior	female	27	abdominal pain	CRC 3A	thymolipoma	38 × 26 × 15 cm	2320	7706	median sternotomy *enbloc* resection	none
Rahman, S.M.T. et al., 2022 [20]	case report	anterior	male	46	dry cough, periodic shortness of breath, and chest heaviness	CRC 3A	lipoma	24 × 16 × 17 cm	5400	3394	median sternotomy *enbloc* resection	none
Daniel, V.C. et al., 2023 [21]	case report	anterior	male	74	no symptoms	CRC 2	thymolipoma and 2 thymomas	23.2 × 19.2 × 6.7 cm	1060	1551	not mentioned *enbloc* resection	not mentioned
Gong, L.H. et al., 2023 [9]	case report	anterior	male	23	progressively worsening breathlessness over 6 months, with respiratory insufficiency	CRC 3B	thymolipoma	26 × 20 × 30 cm	7500	8112	right posterolateral thoracotomy and part of 5th rib removed piecemeal resection	- 800 mL blood loss, transfusion of 4 units erythrocyte concentrates and 300 mL plasma - incomplete re-expansion of the right lung
Soleimani, H. et al., 2024 [22]	case report	anterior	male	18	no symptoms	CRC 1	thymolipoma	13 × 23 × 15 cm	-	2332	right thoracotomy *enbloc* resection	not mentioned
Webb, A.J. et al., 2017 [23]	case report	middle	male	32	worsening intermittent chest pain (6 months), progressive dysphagia, cough, and dyspnea	CRC 3B emergency presentation due to chest pain	solitary fibrous tumor	17.5 × 15 × 8 cm	910	1092	not mentioned	not mentioned
Collaud et al., 2023 [10]	case series (4 cases)	middle	male	41	no symptoms	CRC 1	esophagus leiomyoma	8.2 cm diameter	-	287	posterolateral thoracotomy *enbloc* resection	none
			male	33	cough and sudden decline in exercise capacity	CRC 3A	synovial sarcoma	initially 8.4 cm in diameter, 2.5 × 4 × 4 cm after chemotherapy	-	21	posterolateral thoracotomy tumor debulking (hearth infiltration)	none
			female	70	increasing dyspnea over the last 6 months	CRC 3A	leiomyosarcoma	9 cm diameter	-	379	right posterolateral thoracotomy *enbloc* resection	none
			male	53	chest pain, weight loss, and night sweats	CRC 3A	undifferentiated round cell sarcoma	12 cm in diameter	-	899	median sternotomy and extended resection under cardiopulmonary bypass *enbloc* resection	none
Arras-Martinez et al., 2015 [24]	case report	posterior	male	68	atrial flutter, dyspnea, and dysphagia	CRC 3B emergency presentation (atrial flutter)	liposarcoma	17.5 × 13 × 14 cm	1009	1656	right posterolateral thoracotomy *enbloc* resection tumor and right lower lobe and esophagus muscular outer layer	none
Kandakure, P.R. et al., 2018 [25]	case report	posterior	female	50	severe dyspnea and dysphagia	CRC 3B	liposarcoma	42 × 25 × 10 cm	6000	5460	left posterolateral thoracotomy *enbloc* resection	none
Savu, C. et al., 2020 [26]	case report	posterior	male	60	diminished tolerance of physical activity and mild dyspnea	CRC 3A	schwannoma	20.5 × 12.5 × 9 cm	1830	1199	right anterolateral thoracotomy *enbloc* resection	none
Ataya, J. et al., 2023 [27]	case report	posterior	male	52	dyspnea upon exertion, dry cough, lethargy, and weight loss	CRC 3A	liposarcoma	25 × 10 × 8 cm	2250	1040	right thoracotomy *enbloc* resection	none
Huang, H.Y. et al., 2024 [28]	case report	posterior	female	56	exertion dyspnea, acute respiratory failure requiring intubation in the emergency department	CRC 3B emergency (acute respiratory failure)	schwannoma	16.5 × 12.5 × 12.0 cm	1359	1287	posterolateral thoracotomy *enbloc* resection	pneumonia and recurrent respiratory failure requiring reintubation

## Data Availability

The data supporting the findings of this study are available from the corresponding author upon reasonable request. Due to ethical considerations, the data are not publicly accessible.
